# The diabetes epidemic and its implications for eye health

**Published:** 2015

**Authors:** David Cavan

**Affiliations:** Director of Policy & Programmes: International Diabetes Federation, Brussels, Belgium. **David.Cavan@idf.org**

**Figure:**
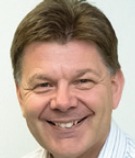
David Cavan

The world is facing an unprecedented epidemic of diabetes. The International Diabetes Federation (IDF) estimates that there are 415 million adults living with diabetes worldwide. No country is immune from this epidemic: 70% of people with diabetes live in low- and middle-income countries.

Type 2 diabetes accounts for over 90% of all cases. The increase in type 2 diabetes is associated with modern-day lifestyles, characterised by unhealthy eating (foods high in sugar, salt and fat), physical inactivity and increasing obesity.

Diabetes causes high levels of glucose (a form of sugar) in the bloodstream. Over time, this damages blood vessels, with devastating effects in many different parts of the body, leading to heart attacks, stroke, foot amputations, kidney failure and blindness. It is these complications of diabetes that lead to premature death in so many. IDF estimates that, in 2015, five million people died from causes associated with diabetes. That is more than all the deaths from malaria, tuberculosis and HIV combined. The complications of diabetes also account for the staggering cost of treating diabetes, estimated at over US $670 billion dollars a year.

**‘Nearly half of all people with diabetes don't know they have the condition’**

Diabetic retinopathy (DR) is caused by damage to retinal blood vessels. It is estimated that one third of people with diabetes have DR, and that up to a third of them have impaired vision. Although advanced DR can lead to blindness, the early stages are entirely asymptomatic. It is therefore essential that everyone with diabetes has their eyes examined for DR, ideally every year.

**Figure:**
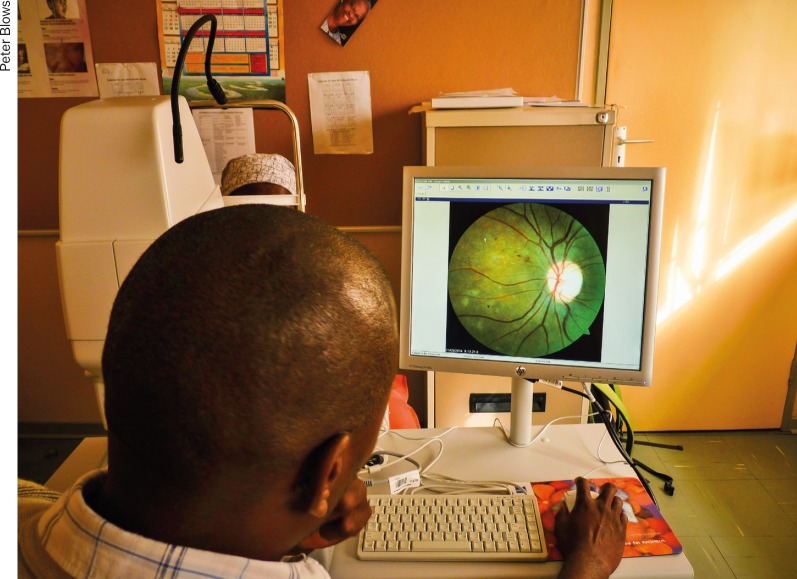
Nurse Baele Fidzani grades an image at Donga Diabetes Centre, Francistown. BOTSWANA

Nearly half of all people with diabetes don't know they have the condition, so the damage to their eyes progresses to an advanced stage before there is an opportunity to prevent vision loss. This is tragic, as the risk of developing DR and vision loss can be reduced by keeping blood glucose, cholesterol and blood pressure as near normal levels as possible.

We need to build health systems that will help people to achieve good control of their diabetes through lifestyle changes and medication where required, and that will provide regular retinal screening to detect DR in its early stages, as well as laser or anti-VEGF treatment to prevent blindness. As most cases of type 2 diabetes can be prevented, there is also an urgent need to promote policies that support healthy lifestyles.

Unfortunately, we are far from achieving this ideal. Too many people already have advanced retinopathy at the time they are diagnosed with diabetes. Too many people with diabetes are not aware of the risk of blindness, and too many of those who see a doctor for their diabetes do not have their eyes examined.

## Understanding the challenges: the DR Barometer project

The DR Barometer was a global project undertaken by IDF, the International Association for the Prevention of Blindness (IAPB), the International Federation on Ageing (IFA) and the New York Academy of Medicine. It collated the experiences of 3,590 people with diabetes and 1,451 health professionals from 41 countries across the globe. The preliminary findings were released at the EURETINA meeting in Nice in September 2015 and provided revealing insights.

One in five people with diabetes were not aware that diabetes could affect their vision. Of those who were aware, many reported that they did not know why, nor what they could do to prevent it. This highlighted the need for better education of people with diabetes about the risks of DR, and the importance of maintaining good blood glucose control.

**‘One in five people with diabetes were not aware that diabetes could affect their vision’**

One in four people with diabetes had not had their eyes examined in the previous two years. Two significant barriers were identified: the long wait times for an appointment and the high cost of the examination. This emphasises the need for accessible and affordable screening to be available to everyone with diabetes.

Over half of all health professionals did not have access to educational materials for patients. A similar proportion had no written protocol for the detection and management of DR. Nearly one in four eye specialists reported that they had received no training in the management of DR and 40% reported that there was poor integration of diabetes and eye care services. Until these fundamental deficiencies are addressed, progress in preventing vision loss in diabetes will be very slow.

## What is being done to improve the situation?

Several organisations and initiatives are working hard to address diabetes and DR.

One of the key aims of IDF is the prevention of type 2 diabetes. It is active at the global and national levels, advocating that governments introduce policies to increase access to fresh, healthy foods and clean drinking water, and reduce consumption of unhealthy foods and sugar-sweetened beverages, which increase the risk of type 2 diabetes. In 2015, the World Health Organization (WHO) issued new recommendations to limit sugar consumption to no more than 5% of a person's daily energy intake. In response, IDF published its Framework for Action on Sugar that detailed twelve actions to reduce consumption of sugar in the general population.

IDF also aims to improve the care of people with diabetes. In 2013, IDF and The Fred Hollows Foundation formed a partnership with the aim of improving the eye health of people with diabetes. The first outcome of this collaboration is *Diabetes Eye Health: A Guide for Health Professionals.* The guide includes practical information for primary health professionals to support them in discussing diabetes management for good eye health and detecting DR in people with diabetes. The guide will be published in the six UN languages (English, French, Spanish, Arabic, Chinese and Russian) and is available for download free of charge via the IDF website. It is hoped that this guide will help fill the gaps identified in the Barometer study and provide a framework to guide individual health professionals and local health systems in structuring screening and treatment services for DR. Further details of the Guide are presented in the panel below.

While some aspects of training in DR require hands-on instruction, much can be learnt online, bye-learning, and IDF is planning to develop interactive modules to make available the latest information on screening and treatment for DR. This will help health professionals to provide accurate information to people with diabetes from the moment they are diagnosed. IDF has recently launched the first of a two-part introductory module for non-specialist health professionals, aimed at equipping everyone involved in the care of people with diabetes with the knowledge they need to provide basic lifestyle advice (see Useful Resources below).

The Queen Elizabeth Diamond Jubilee Trust (QEDJT), through the Commonwealth Eye Health Consortium, has enabled the formation of the Diabetic Retinopathy Network (DR-NET) in 10 Commonwealth countries, whereby existing VISION 2020 LINKS between UK and overseas eye departments share learning on DR screening and treatment. In addition, DR-NET facilitates improved coverage of fundus cameras and screening databases and also works with Ministries of Health to implement national frameworks for diabetic retinopathy.

Further initiatives are planned in 2016 to promote better screening for, and treatment of, DR. There are excellent examples, such as the UK Retinal Screening programme, which demonstrate that effective population-based screening can be achieved. The challenge is to see this replicated elsewhere, in different national health systems and with different levels of resources. In order to build the evidence base for a structured approach to managing DR in low-resource settings, The Fred Hollows Foundation have partnered with the QEDJT and others to implement trials of models of care that integrate eye health into diabetes care in Pakistan, Bangladesh and the Pacific Islands.

**Diabetes Eye Health: a guide for health professionals**

**Figure:**
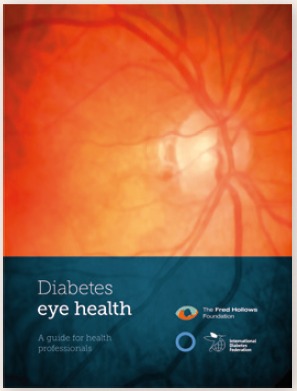

The rising number of people developing diabetes worldwide means there will be an increasing number of people with diabetic eye disease. Early detection and treatment of diabetic retinopathy (DR) is needed to reduce the burden of vision loss on individuals, their caregivers and society.

Specialised eye health practitioners have an important role to play in addressing DR; however, as they are a relatively limited resource, their focus should be on treatment. The support of primary health practitioners – the general practitioners, family doctors, nurses, endocrinologists and others who manage the primary care of people with diabetes – is therefore vital for the early diagnosis and timely management of diabetic eye diseases.

Many people with diabetes – as well as many health professionals – are unaware of the critical need to undergo regular eye examinations. Primary health professionals, through their routine care of people with diabetes, are the ones most likely to have the opportunity to screen patients and to educate and support them to manage their diabetic eye disease. They can also facilitate the timely referral of patients to eye specialist services for treatment to reduce sight loss.

It is with this in mind that the International Diabetes Federation (IDF) and The Fred Hollows Foundation launched *‘Diabetes Eye Health: A Guide for Health Professionals’* last year. The purpose of the guide is to educate and inform primary health care professionals about diabetic eye diseases and to show them, in very practical ways, what they can do to address the rising prevalence of diabetic-related eye disease, particularly diabetic retinopathy.

The three key actions by health professionals to manage eye health in people with diabetes are:

1. Helping people with diabetes to optimise their control of blood glucose, blood pressure and blood lipids in order to slow down the progression of diabetic retinopathy.
2. Ensuring that people with diabetes have regular eye examinations and timely treatment when required.
3. Educating and supporting people with diabetes to manage their own eye health and their diabetes.

**We would like to encourage readers concerned about DR to make contact with the relevant primary health care workers in your area. Share with them the key messages in this article, offer information about *Diabetes Eye Health: A Guide for health professionals* and inform them about any diabetic eye disease services available in your area.**

Diabetes Eye Health: A Guide for Health Professionals. Available for download from **www.idf.org/sites/default/files/Diabetes_Eye_Health.pdf**

## What can eye health professionals do to improve the situation?

Unless we act now to develop prevention, screening and treatment services for DR, we face the prospect of nearly 40 million people experiencing vision loss from diabetes, all of whom will require multiple review and treatment visits. This will be a significant burden on top of ophthalmologists' existing work load – even more so in low- and middle-income countries where there are very few of these specialists.

DR is preventable and blindness from DR is avoidable, but only if there is close collaboration between diabetes and eye health professionals at local, national and global level. In order to promote this, IDF and The Fred Hollows Foundation are exploring the creation of a global ‘DR Alliance’ to raise awareness of DR and to take the lead in recommending solutions to help address it.

In the meantime, we encourage all eye health professionals to improve the situation in their area in the following ways:

- Set an example by adopting a healthy lifestyle.
- Provide basic lifestyle advice to all patients, whether or not they have diabetes. This will help to prevent new cases of type 2 diabetes and help prevent DR in those with diabetes.
- Ensure patients with diabetes are being appropriately monitored by a primary care or specialist diabetes physician. This is especially important for those with DR.
- Build links with local diabetes professionals to develop reliable care pathways for patients with DR and set up a screening (fundus) camera in the diabetes clinic, so it is easily accessed by the target population.
